# PI(4,5)P_2_ Imaging Using a GFP Reporter in Living Cells

**DOI:** 10.21769/BioProtoc.5336

**Published:** 2025-06-05

**Authors:** Mariam Alkandari, Christopher R. McMaster, Mahtab Tavasoli

**Affiliations:** Department of Pharmacology, Dalhousie University, Halifax, NS, Canada

**Keywords:** PI(4, 5)P_2_, Phospholipid, Membranes, Fluorescence assay, Localization, Confocal microscopy

## Abstract

Phosphatidylinositol 4,5-bisphosphate [PI(4,5)P_2_] is a phospholipid enriched on the cytoplasmic leaflet of the plasma membrane, where it plays important roles in membrane trafficking and cytoskeletal dynamics through proteins that directly bind to it. PI(4,5)P_2_ can be metabolized to other phosphorylated forms of phosphatidylinositol to regulate numerous processes such as cell growth and development. PI(4,5)P_2_ can also be hydrolyzed to generate the second messengers diacylglycerol (DAG) and inositol triphosphate (IP_3_). Altered metabolism or mislocalization of PI(4,5)P_2_ can perturb one or more of its functions and contribute to disease states. Here, we present a protocol to visualize and quantify the localization of PI(4,5)P_2_ in live cells. The protocol uses a highly specific PI(4,5)P_2_ protein binding domain coupled to enhanced green fluorescence protein (PH-PLCD1-GFP), enabling localization and quantification of cytosol-facing PI(4,5)P_2_ to be determined. Localization and quantification of the PH-PLCD1-GFP, PI(4,5)P_2_ specific probe, is enabled by fluorescence imaging and confocal microscopy. This approach can be used to study the dynamics of PI(4,5)P_2_ localization temporally in live cells under both physiological and pathological conditions.

Key features

• Protocol for the quantification of PI(4,5)P_2_ membrane localization in live cells.

• Uses the expression of the highly specific PH-PLCD1-GFP, PI(4,5)P_2_ probe, in cells, followed by fluorescence image acquisition using confocal microscopy and subsequent image processing.

• Adaptable to various cell types and experimental conditions.

• Presents detailed instructions for reagent preparation, fluorescence measurement, and quantification.

## Graphical overview


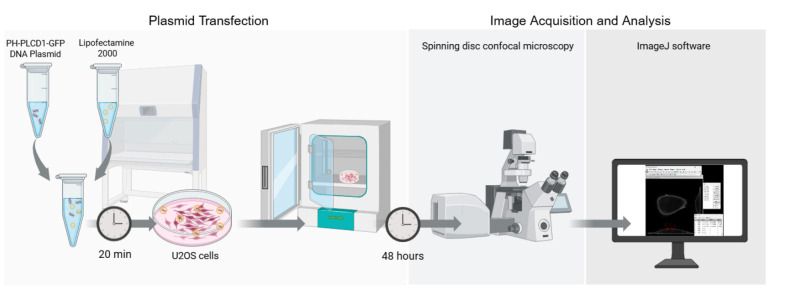


**Graphical overview of the key steps for quantifying PI(4,5)P_2_ localization in living cells**

## Background

Phosphatidylinositol 4,5-bisphosphate [PI(4,5)P_2_] is a phospholipid that belongs to the phosphoinositide family. Phosphoinositides are phosphorylated derivatives of phosphatidylinositol that can be further phosphorylated at different positions on the inositol ring to generate a variety of distinct lipid signaling molecules [1,2]. PI(4,5)P_2_ is a phosphoinositide that is preferentially localized to the inner leaflet of the plasma membrane [3–5]. PI(4,5)P_2_ can bind to numerous proteins, often through a distinct PI(4,5)P_2_ binding domain, with exemplars including Pleckstrin homology (PH) domain, Phox (PX) homology domain, Postsynaptic Density-95 (PSD-95)/Discs Large (Dlg)/Zonula Occludens-1 (PDZ), Protein Kinase C conserved Region 2 (C2), Src Homology 2 (SH2), Plant Homeodomain (PHD), and Myristoylated Alanine-Rich C-Kinase Substrate (MARCKS) domains [2,6–10]. This binding often aids in the localization of a protein to the plasma membrane, where they regulate numerous processes such as membrane trafficking and cytoskeletal dynamics [1,5,7–11]. PI(4,5)P_2_ also serves as a substrate for phospholipase Cs that convert PI(4,5)P_2_ to diacylglycerol and inositol triphosphate (IP_3_). Diacylglycerol can itself recruit proteins to membranes, including members of the protein kinase C family, while IP_3_ releases Ca^2+^ from the endoplasmic reticulum [12,13]. Perturbation of the level or localization of PI(4,5)P_2_ can have profound effects on cell dynamics and cell signaling and contribute to disease states [14–16].

Numerous phosphoinositide binding domains have varying affinities and specificities for each phosphoinositide [2,3,17]. The PH domain of phospholipase C delta (PH-PLCD1) has high affinity and specificity for PI(4,5)P_2_, and a chimeric PH-PLCD1-GFP fusion is an accurate reporter for PI(4,5)P_2_ localization [5,6,11,17,18]. This interaction has been validated in multiple species and cell types, including mammalian cells, *Xenopus* oocytes, *Drosophila*, and yeast, supporting its broad applicability [19–22]. Our optimized protocol allows for the visualization of PI(4,5)P_2_ and enables the quantification of the ratio of plasma membrane to intracellular PI(4,5)P_2_. Using the PH-PLCD1-GFP fusion allows for real-time high-resolution tracking of PI(4,5)P_2_ in live cells. This protocol serves as a comprehensive step-by-step guide providing a standardized approach to quantify PI(4,5)P_2_ plasma membrane localization.

## Materials and reagents

**Biological materials**

1. U20S cells (ATCC, HTB-96)

2. PH-PLCD1-GFP plasmid (Addgene, plasmid #51407; map available at: https://www.addgene.org/51407)

**Reagents**

1. Lipofectamine 2000 (Invitrogen^TM^, catalog number: 11668019)

2. Opti-MEM (Gibco, catalog number: 31985070)

3. DMEM, high glucose (Gibco, catalog number: 11965092)

4. Fetal bovine serum (FBS) (Gibco, catalog number: A3160502)

5. Antibiotic-antimycotic (AA) (100×) (Gibco, catalog number: 15240062)

**Laboratory supplies**

1. Two-chamber Lab-Tek^®^ II chambered German coverslip system (Thermo Fisher Scientific, catalog number: 155379)

2. 1.5 mL Eppendorf tubes (Eppendorf, catalog number: 0540294)

3. Eppendorf Research^®^ pipettes, 0.5–10 μL, 10–100 μL, 20–200 μL, 100–1,000 μL (Eppendorf, catalog number: 3123000942)

4. Biosphere^®^ plus filter tip, 1000 μL, 200 μL, 100 μL, 10 μL (Sarstedt)

## Equipment

1. Zeiss Axio Observer Z.1 spinning disk confocal microscope (Carl Zeiss AG)

2. Automated cell counter (Bio-Rad, model: 1450102)

3. Cell counting dual-chambered slides (Bio-Rad, catalog number: 1450011)

4. Tissue culture incubator, 37 °C, 5% CO_2_ (Eppendorf, model: CellXpert^®^ C170i)

5. Tissue culture hood, class II biosafety cabinet (Bio-Klone, model: BK-2-4)

## Software and datasets

1. ZEISS ZEN (version 3.1; Carl Zeiss Microscopy GmbH, Jena, Germany)

2. Fiji (ImageJ-win64; version 1.54; https://imagej.net/software/fiji/downloads)

3. Microsoft Excel (version 2502; Microsoft Corporation, Redmond, WA, USA)

4. GraphPad Prism (version 10; GraphPad Software, San Diego, CA, USA)

## Procedure

**A. Transfection of U2OS cells with PH-PLCD1-GFP**

All the following steps are performed in a Class II biosafety cabinet under sterile conditions.

1. Seed U2OS cells in 1 mL of complete media (DMEM supplemented with 10% FBS and 1% AA) in a 2-well chamber slide system at a density of 50,000 cells per well one day prior to transfection.

*Note: The size of each well in a 2-well chamber slide system is 4.2 cm^2^. If a different well format is used, volumes/concentrations may be upscaled or downscaled accordingly.*

2. For cell counting, load 10 μL of cell suspension into each chamber of the dual-chambered counting slide and insert into the Bio-Rad automated cell counter, which automatically provides total cell concentration in cells/mL.

3. On the day of transfection, change media to AA-free DMEM media supplemented with 10% FBS (1 mL per well).

4. In a 1.5 mL Eppendorf tube, dilute PH-PLCD1-GFP DNA plasmid in Opti-MEM to achieve a concentration of 0.5 μg in 75 μL of Opti-MEM per well.

5. In another 1.5 mL Eppendorf tube, dilute Lipofectamine 2000 in Opti-MEM to achieve a concentration of 3 μL of Lipofectamine 2000 in 75 μL of Opti-MEM per well.

6. Add diluted PH-PLCD1-GFP DNA (0.5 μg in 75 μL of Opti-MEM) to diluted Lipofectamine 2000 (3 μL in 75 μL of Opti-MEM) and gently mix by pipetting. Incubate at room temperature for 20 min.

7. Add 150 μL of PH-PLCD1-GFP DNA-Lipofectamine complex mixture dropwise to each well and incubate cells at 37 °C with 5% CO_2_ for 24 h.

8. Twenty-four hours post-transfection, change transfection media to fresh complete DMEM media with AA and incubate cells at 37 °C with 5% CO_2_ for an additional 24 h to allow peak expression of the GFP reporter, consistent with the general transfection guidelines. The post-transfection time frame for optimal reporter expression can be optimized based on the cell type being studied.

9. Following 48 h post-transfection, cells are ready for imaging. The robust expression of the GFP-tagged reporter in over 80% of U2OS cells confirmed successful transfection.

**B. Imaging U2OS cells transfected with PH-PLCD1-GFP**

Images of U2OS cells transfected with PH-PLCD1-GFP are acquired using a Zeiss Axio Observer Z.1 spinning disk confocal microscope at 100× magnification in a temperature-controlled chamber maintained at 37 °C with 5% CO_2_ and ambient O_2_ and humidity following the steps described below.

*Notes:*

*1. While not strictly required, a spinning disc confocal microscope was used for live cell Z-stack imaging due to its ability to capture rapid transient dynamics through faster acquisition and its lower phototoxicity.*

*2. By using the lowest laser power necessary to acquire high-quality images, along with a spinning disk confocal system, photobleaching was minimized and not observed under the conditions used.*

*3. Imaging was carried out over a period of approximately 10–20 min, during which pH was preserved by using CO_2_-buffered imaging medium. No replenishment of medium was required during the acquisition period as the imaging time was kept short and environmental parameters (e.g., temperature and CO_2_ levels) were well controlled.*

1. Set up the parameters for image acquisition as follows: laser intensity at 20%, exposure at 65 m/s, and EM gain at 50. Select cells that are well defined and not in proximity to other cells within the microscope field.

**Figure 1.**
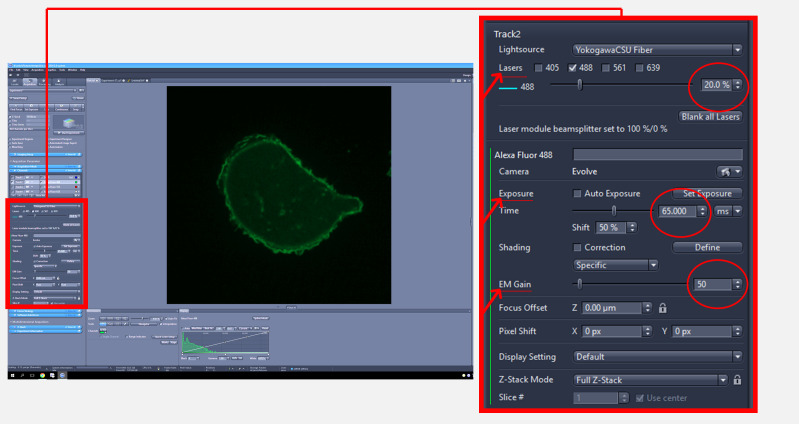
Zeiss imaging software interface for adjusting image acquisition settings. The full screen view (left) displays the overall interface, with a red square indicating the region that is magnified in the inset (right) to show laser intensity, exposure, and EM gain settings used for image acquisition.

*Note: These settings can be used as a starting point and may require adjustment to optimize the signal to background ratio based on cell type, reporter expression, and microscope configuration (Figure 1).*

**Figure 2.**
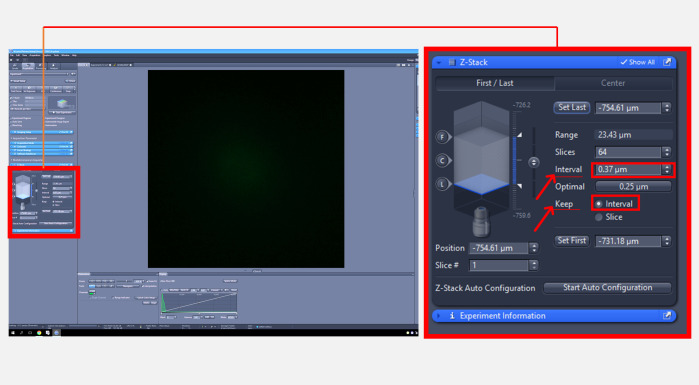
Zeiss imaging software interface for adjusting Z-stack image acquisition settings. The full screen view (left) displays the overall interface, with a red square indicating the region that is magnified in the inset (right) to show Z-stack image acquisition settings.

2. Once a cell has been selected, acquire z-stack images using a 488 nm excitation wavelength and an emission filter at 517 nm while keeping a constant interval (0.36 μm) between each focal plane (Figure 2).

*Note: Image acquisition may be carried out in a blinded manner to minimize bias.*

**Figure 3.**
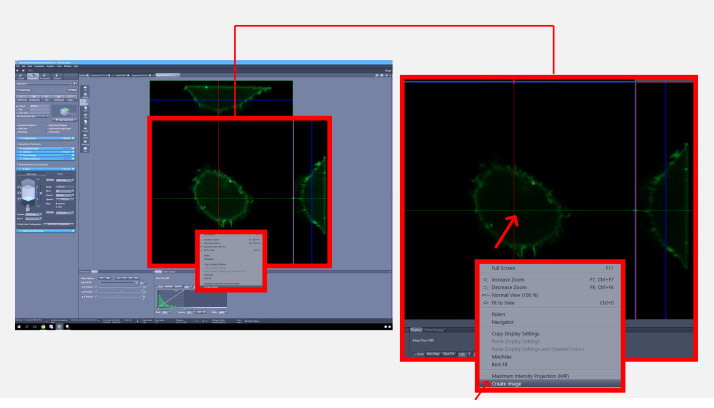
Zeiss imaging software interface for an orthogonal view of the cell generated from the Z-stack. The full screen view (left) displays the overall interface, with a red square indicating the region that is magnified in the inset (right) to show the intersection point at the center of the cell to generate an orthogonal view.

**Figure 4.**
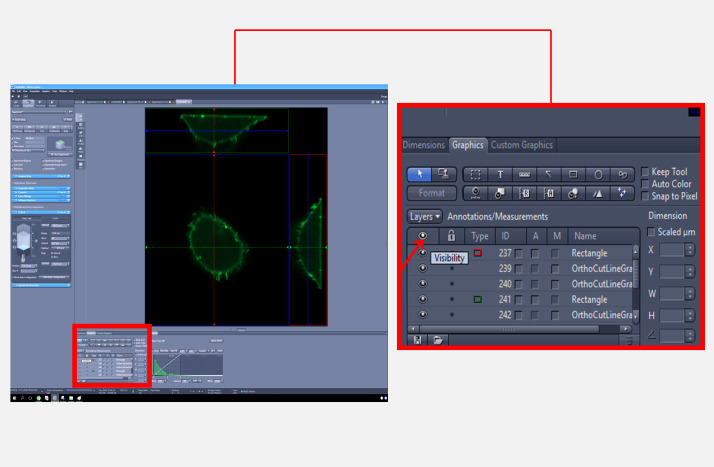
Zeiss imaging software interface for an orthogonal view of the cell generated from the Z-stack. The full screen view (left) displays the overall interface, with a red square indicating the region that is magnified in the inset (right) to demonstrate how to hide axis lines by unchecking the visibility option in the graphics section.

3. After acquiring the Z-stack image, display the image in orthogonal (ortho) mode, position the intersection point at the center of the cell, and create an image from the view (Figure 3).

4. In the graphics section, uncheck the visibility option to hide the lines and save the image in TIFF format (Figure 4).

**C. Quantification of the plasma membrane to cytosol ratio of PH-PLCD1-GFP**

1. Open images in the FIJI ImageJ software and convert them to grayscale (8-bit or 16-bit).

*Notes:*

*1. Quantification by FIJI may be carried out in a blinded manner to minimize bias.*

*2. If your image is too bright or too dark, you may need to adjust the contrast/brightness.*

2. Go to the ROI Manager by clicking *Analyze* > *Tools* > *ROI manager*.

3. Select the *rectangle* tool from the *Toolbar* to draw the first square on the image.

*Note: Due to variability in cell size across cell types, the dimensions of the rectangular ROI were not strictly prespecified. Instead, the ROI size was adjusted to ensure appropriate sampling of either cytosolic or membrane signal, while maintaining consistency in size across ROIs within the same imaging field and across the same experiment. The ROI was chosen to be small enough to focus on subcellular regions of interest, rather than encompassing the entire cell.*

**Figure 5.**
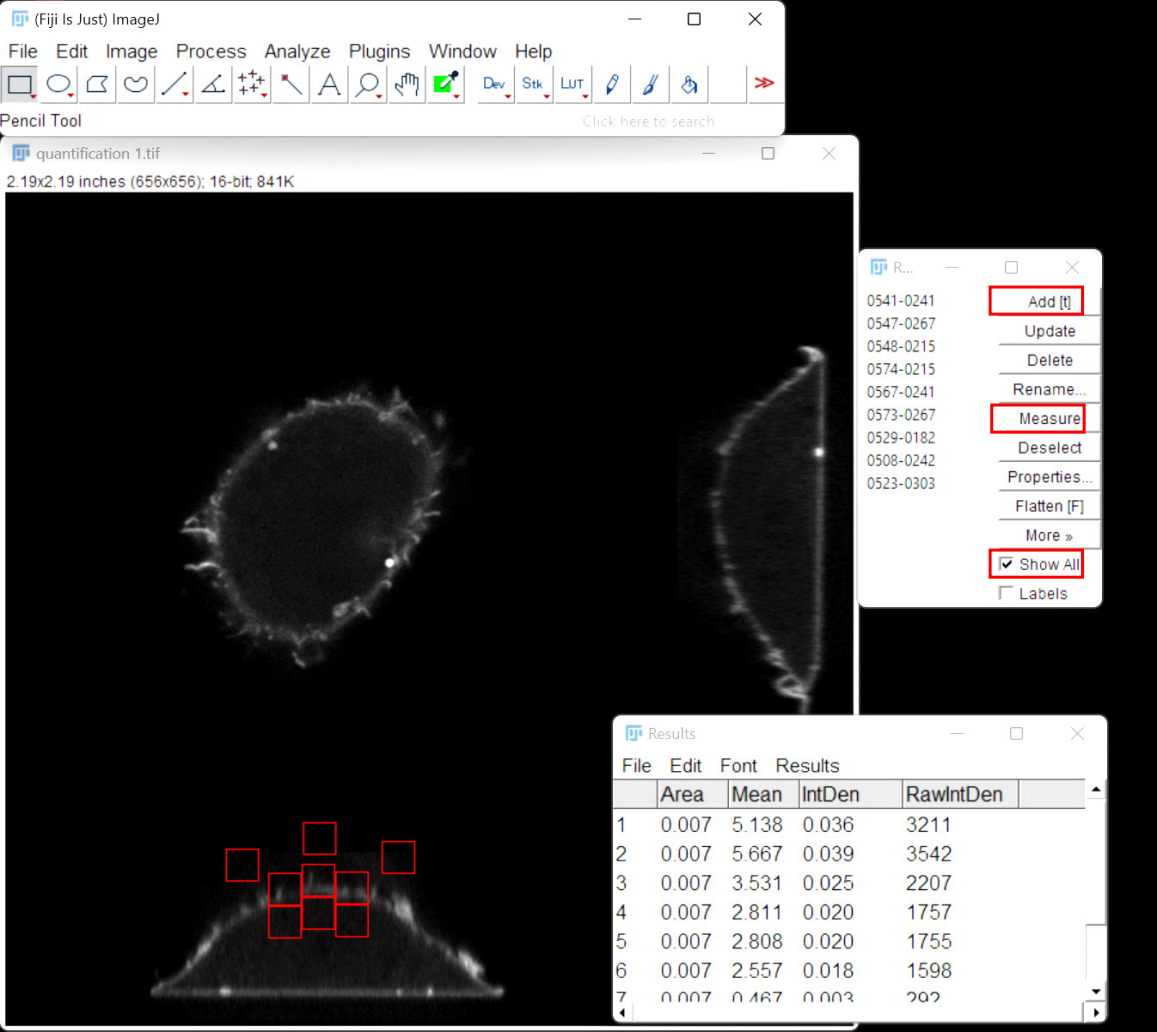
ImageJ interface showing placement of ROIs corresponding to the plasma membrane, cytosol, and background. Mean fluorescence intensities were measured within each ROI to calculate the plasma membrane to cytosol intensity ratio.

4. When the rectangle is in the desired position, click *Add [t]* in the ROI manager.

5. Enable *show all* on the ROI manager to view all saved ROIs.

6. Draw three equal squares on the dorsal plasma membrane (cell apex and immediately left and right) and another three identical squares below these in the cytosol, and three random squares outside the cell (background) (Figure 5).

*Notes:*

*1. For the squares positioned immediately to the left and right of the apex, place the squares at locations that encompass most of the membrane within each square.*

*2. Membrane intensity was measured at the apical region of the cell to standardize the sampling location. While the GFP signal may vary along the membrane, consistent apical sampling ensured comparability across conditions.*

*3. A separate membrane marker was not used, as the PH-PLCd1-GFP reporter provides sufficient and specific plasma membrane labeling due to its selective binding to PI(4,5)P_2_.*

**Figure 6.**
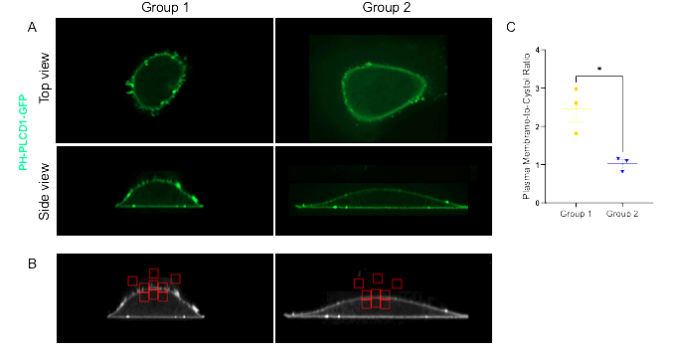
Quantification of PH-PLCD1-GFP localization. A. acquired microscopy images of U2OS cells transfected with PH-PLCD1-GFP. B. Processed images from panel A using FIJI/ImageJ following steps outlined in section C to quantify plasma membrane to cystol fluorescence intensity ratio. C. Quantification dorsal plasma membrane: cytoplasmic PH-PLCD1-GFP ratio. Bars show mean± SEM. Statistical significance was determined using t-test, *P < 0.05.

7. Once the nine squares are added to the ROI manager, click *measure* in the ROI manager and export results (Figure 5).

8. Calculate the mean intensity from the three background ROIs and subtract this value from each of the individual mean intensity measurements for the plasma membrane and cytosol.

9. Divide the mean intensity of each of the three squares on the dorsal plasma membrane (one on the apex and two immediately adjacent) by the mean intensity of the cytosolic ROI directly below it. The final plasma membrane to cytosol ratio of PH-PLCD1-GFP per cell is the average of three individual membrane to cytosol ratios. An Excel sheet with representative calculations is included in the supplementary materials (Supplementary file 1).

10. By following steps C1–9, the plasma membrane to cytosol ratio of PH-PLCD1-GFP was quantified for two images to demonstrate the quantification approach described in this protocol (Figure 6).

## Data analysis

Data analysis is performed to study the subcellular localization of PI(4,5)P_2_ by quantifying the plasma membrane to cytosol ratio of PH-PLCD1-GFP. The steps for ratio calculation are outlined in section C and illustrated in the supplementary Excel sheet. Quantification is performed for 10–20 cells for each experimental group from three independent experiments. (*Note: Figure 6 includes data from a single cell per group and is intended for illustrative purposes only.*) For comparison of two groups, a two-tailed t-test is used. Comparison involving more than two groups is performed using one-way ANOVA followed by Tukey’s multiple comparison test. A threshold of P < 0.05 was used to determine statistical significance. Results may be communicated through representative images together with quantification of the PH-PLCD1-GFP plasma membrane to cytosol fluorescence intensity ratio (Figure 6A, C). Quantitative analysis may be shown as a bar graph with a scatter plot or scatter blot with a bar indicating the mean. Statistical analysis and graph generation were performed using GraphPad Prism.

## Validation of protocol

This protocol or parts of it has been used and validated in the following research article:

- Tavasoli et al. [23]. Defects in integrin complex formation promote *CHKB*-mediated muscular dystrophy. *Life Science Alliance* (Figure 4A–C and G–I).

## General notes and troubleshooting

**General notes**

1. Seeding density and DNA:Lipofectamine/DNA/cell ratio may require further optimization if a different cell type is used.

**Troubleshooting**

Problem 1: Transfection efficiency may vary dramatically across cells.

Possible cause: Following our steps for U2OS, the transfection efficacy was uniform across cells, and lipofectamine toxicity was not observed. However, transfection efficiency differences and lipofectamine toxicity are not uncommon in cells, which may result in variability in signal intensity across cells.

Solution: 1: Transfection experiments should be optimized for the cell line under study for optimal plasmid uptake and minimal toxicity. A helpful reference provided by Thermo Fisher Scientific can be used as a starting point for transfection optimization (https://www.thermofisher.com/ca/en/home/references/gibco-cell-culture-basics/transfection-basics/applications/plasmid-transfection/optimizing-plasmid-dna-transfection.html). Solution 2: Given that a ratio of signal between two areas (plasma membrane vs. intracellular) within the same image field is to be calculated, imaging settings can be adjusted for each field to increase or decrease the signal, as these adjustments should not impact the relative ratio.

Problem 2: GFP PH-PLCD1 reporter does not show expected membrane localization or displays unexpected intracellular distribution.

Possible cause: The reporter may not be binding specifically to PI(4,5)P_2_ due to suboptimal experimental conditions. Alternatively, the reporter itself may not be functioning properly.

Solution: To verify that the reporter is working as intended, several controls and validation steps should be included:

1. Positive control: Co-transfect the reporter with a known PI(4,5)P_2_-stimulating construct (e.g., mCherry- PIP5K1α) or activate pathways that elevate PI(4,5)P_2_ (e.g., through GPCR or RTK activation) [24,25]. This should enhance membrane localization of the reporter. When using signaling pathway activation, it is a good idea to ensure that the selected pathway is supported by the literature to be expressed or functional in the studied cell line.

2. Negative control: Use a mutant PH domain that cannot bind PI(4,5)P_2_ or apply conditions that deplete PI(4,5)P_2_ (e.g., overexpression of phosphoinositide phosphatases) [26,27]. This should abolish or reduce PI(4,5)P_2_ membrane localization to confirm the specificity of the (WT) PH domain construct.

3. Membrane enrichment quantification: Measure membrane-to-cytosol fluorescence intensity ratios to assess reporter enrichment. Consistent membrane localization in control conditions supports proper reporter functionality.

Problem 3: Photobleaching observed during imaging.

Cause: High laser power or prolonged exposure times during image acquisition.

Solution: Use the lowest laser power necessary for acquiring high-quality images. Spinning disk confocal systems are preferred as they reduce phototoxicity and minimize photobleaching due to rapid image acquisition. Shorten exposure times and avoid repeatedly imaging the same field to reduce fluorescence loss.

Problem 4: Cells do not achieve optimal confluency before transfection.

Cause: Inconsistent seeding or over- or under-seeding of cells.

Solution: Seed cells at the recommended density (as outlined in the protocol) and allow them to adhere for at least 16–24 h prior to transfection. Ensure even distribution of cells when seeding to avoid regions of over-confluency, which can affect transfection efficiency and membrane dynamics.

Problem 5: Inconsistent live-cell imaging conditions during acquisition.

Cause: Fluctuations in temperature, CO_2_, or humidity within the live cell chamber.

Solution: Ensure that the live cell imaging chamber is pre-equilibrated to 37 °C with 5% CO_2_ and that humidity levels are maintained. Avoid removing the dish from the incubator for extended periods to prevent changes in cell culture conditions. Always monitor environmental settings throughout imaging.

Problem 6: Long quantification times for large numbers of images.

Cause: Manual quantification of large datasets can be time-consuming.

Solution: Consider using ImageJ/Fiji macros or batch processing tools to streamline quantification. Pre-programmed macros (e.g., using the ROI Manager) can automatically process multiple images and reduce manual effort, especially for high-throughput applications.

## Supplementary information

The following supporting information can be downloaded here:

1. Supplementary file 1. Representative calculations
